# The role of adrenomedullin in the pathogenesis of gastric cancer

**DOI:** 10.18632/oncotarget.18881

**Published:** 2017-06-29

**Authors:** Fuhao Qiao, Jian Fang, Jinfeng Xu, Wenqiu Zhao, Ying Ni, Bufugdi Andreas Akuo, Wei Zhang, Yun Liu, Fangfang Ding, Guanlin Li, Baoguo Liu, Hua Wang, Shihe Shao

**Affiliations:** ^1^ School of Medicine, Jiangsu University, Zhenjiang 212013, Jiangsu, PR China; ^2^ Medical Laboratory, Xintai Hospital of Traditional Chinese Medicine, Xintai 271200, Shandong, PR China; ^3^ School of The Environment and Safety Engineering, Jiangsu University, Zhenjiang 212013, Jiangsu, PR China; ^4^ Nuclear Medicine Laboratory, Taian Jiangong Hospital, Taian 271001, Shandong, PR China

**Keywords:** adrenomedullin, autophagy, gastric cancer, RNA interference, signaling pathway

## Abstract

Adrenomedullin has been shown to be overexpressed in many tumors, including gastric cancer tumors; however, its mechanism of action remains unclear. In this study, we examined the role of adrenomedullin in the pathogenesis of gastric cancer. Using clinical specimens and immunohistochemistry, we found that the expression levels of adrenomedullin and its receptors are inordinately elevated as compared to the adjacent non-tumor gastric tissues. We used siRNA gene silencing, in BGC-823 gastric cancer cell lines, to target adrenomedullin genes, and found that increased adrenomedullin expression results in the proliferation of tumor cells, tumor invasion, and metastasis. Furthermore, we found that under hypoxic conditions, gastric cancer BGC-823 cells exhibit higher expression levels of adrenomedullin and various other related proteins. Our results indicate the involvement of adrenomedullin in microvessel proliferation and partially in the release of hypoxia in solid tumors. Knockdown of adrenomedullin expression, at the protein level, reduced the levels of phosphoprotein kinase B and B-cell lymphoma 2 but increased the levels of cleaved-caspase3 and Bcl 2 associated x protein (Bax). Therefore, we hypothesized siRNA targeting of adrenomedullin genes inhibits various serine/threonine kinases via a signaling pathway that induces cell apoptosis. SiRNA targeting of adrenomedullin genes and green fluorescent control vectors were used to transfect BGC-823 cells, and western blot analyses were used to detect changes in the rates of autophagy in related proteins using confocal laser scanning microscopy. No significant changes were detected. Therefore, the knockdown of adrenomedullin and its receptors may represent a novel treatment strategy for gastric cancer.

## INTRODUCTION

Andrenomedullin (AM) is a ubiquitous peptide, expressed throughout the body, that can cause pheochromocytoma [1]. Gastric carcinoma is the most common type of malignant cancer after lung and liver cancer, and it is the third leading cause of cancer death worldwide [2, 3]. Nearly 40% of patients present with metastatic disease, and approximately 50% present with loco-regional diseases [4]. Recurrence rates are high, and the five-year survival rate, for all stages, is low, ranging from 25% to 30% [4–7]. Despite advances in diagnostic and treatment options, the prognosis of gastric cancer has not improved over the last two decades [8, 9]. Many studies have shown that the level of AM is significantly elevated in various cancerous tumors, including osteosarcomas [10], pancreatic adenocarcinomas [11], and plexiform neurofibromas [12]. AM, a hormone that regulates stem cell differentiation, also regulates many normal activities in the stomach [13]. AM also plays a role in gastric mucosal defense and repair [14, 15]. In endometrial cancer tissues and chromophobe renal carcinomas, AM mRNA levels have been shown to be elevated, whereas protein expression levels were attenuated [16, 17], indicating a putative, complex post-transcriptional regulatory network. In this study, we examined the role of AM in gastric cancer, and analyzed its localization and relationship to the pathogenesis of gastric cancer in clinical samples.

Previous studies have demonstrated that AM plays a pivotal role in several important signaling pathways, including the cAMP, phosphatidylinositol 3-kinase/Akt-dependent, and ERK signaling pathways. AM, via the phosphatidylinositol 3-kinase/Akt-dependent pathway, induces endothelium-dependent vasorelaxation [18], and infusion attenuates myocardial ischemia/reperfusion injury [19]. Furthermore, AM signaling, via the activation of the MAPK/ERK pathway, regulates additional downstream signaling pathways that promote endothelial cell growth and survival [20]. AM up-regulation of Bcl-2, in an autocrine/paracrine manner, also protects malignant cells from hypoxia-induced cell death [21]. Therefore, AM may have a functional in gastric cancer pathogenesis via the activation of a similar signaling pathway in BGC-823 cells transfected with AM siRNA.

Previous studies have shown when tumor cells were exposed to hypoxic conditions, HIF-1αregulated the expression of some genes to aid in the survival of tumor cells [16]. Furthermore, hypoxia-induced expression of AM has been shown to be increased in a variety of tumor cell lines, including multiple myelomas [17], and bladder urothelial cell carcinomas [22]. However, the role of AM under hypoxic conditions has not been described in gastric cancer tumors. Thus, BGC-823 cells were cultured under hypoxic conditions to determine whether they respond via the same hypoxia-induced mechanisms.

Autophagy is an evolutionary conserved catabolic process by which cells degrade and are recycled to maintain cellular homeostasis [23]. Autophagy accompanies apoptosis, via ER-stress mediated mechanisms, in human gastric cancer SNU-16 cells [24]. Several reports have demonstrated an alteration in autophagy by the calcium signalosome in human diseases [23]. Furthermore, it has been shown that AM induces Ca^2+^ mobilization, independent of cAMP levels [25]. Here, we utilize a pcDNA-GFP-LC3 single fluorescent autophagy indicator system to determine the relationship between AM and autophagy.

## RESULTS

### AM and AM receptors are highly expressed in gastric adenocarcinomas

**Figure 1:**
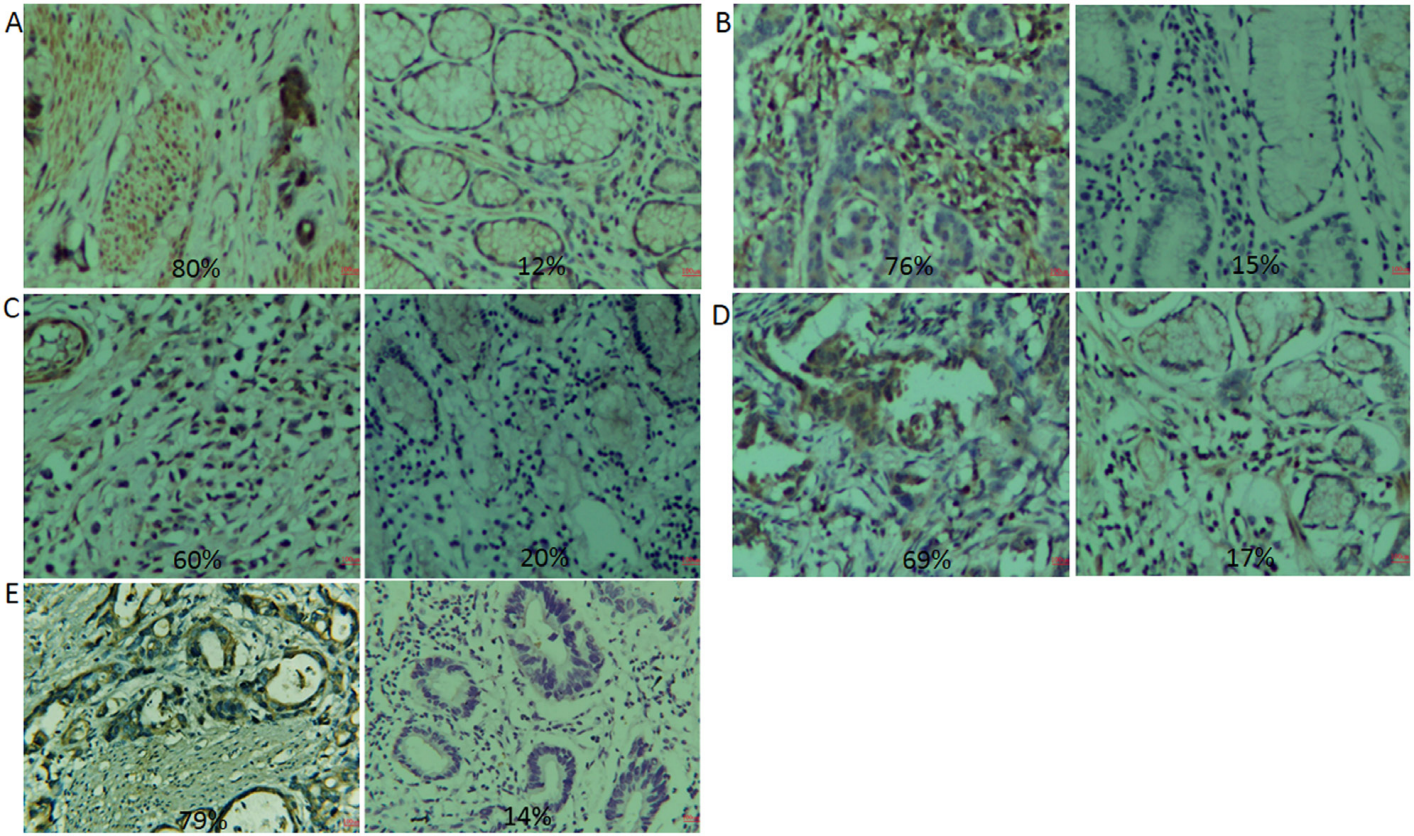
Immunohistochemical staining of tissue specimens The intensity of AM and AM receptor staining in the gastric cancer tissue samples was scored as 0% (negative), 25% (weak), 50% (moderate), 75% (strong), or 100% (strongest) as determined by a pathologist from ADICON CLINICAL LABORATORIES. **(A)** Staining showing the expression of AM (left, gastric cancer tissue; right, adjacent tissue). **(B)** Staining showing the expression of RAMP1 (left, gastric cancer tissue; right, adjacent tissue). **(C)** Staining showing the expression of RAMP2 (left, gastric cancer tissue; right, adjacent tissue). **(D)** Staining showing the expression of RAMP3 (left, gastric cancer tissue; right, adjacent tissue). **(E)** Staining showing the expression of CRLR(left, gastric cancer tissue; right, adjacent tissue).

**Figure 2:**
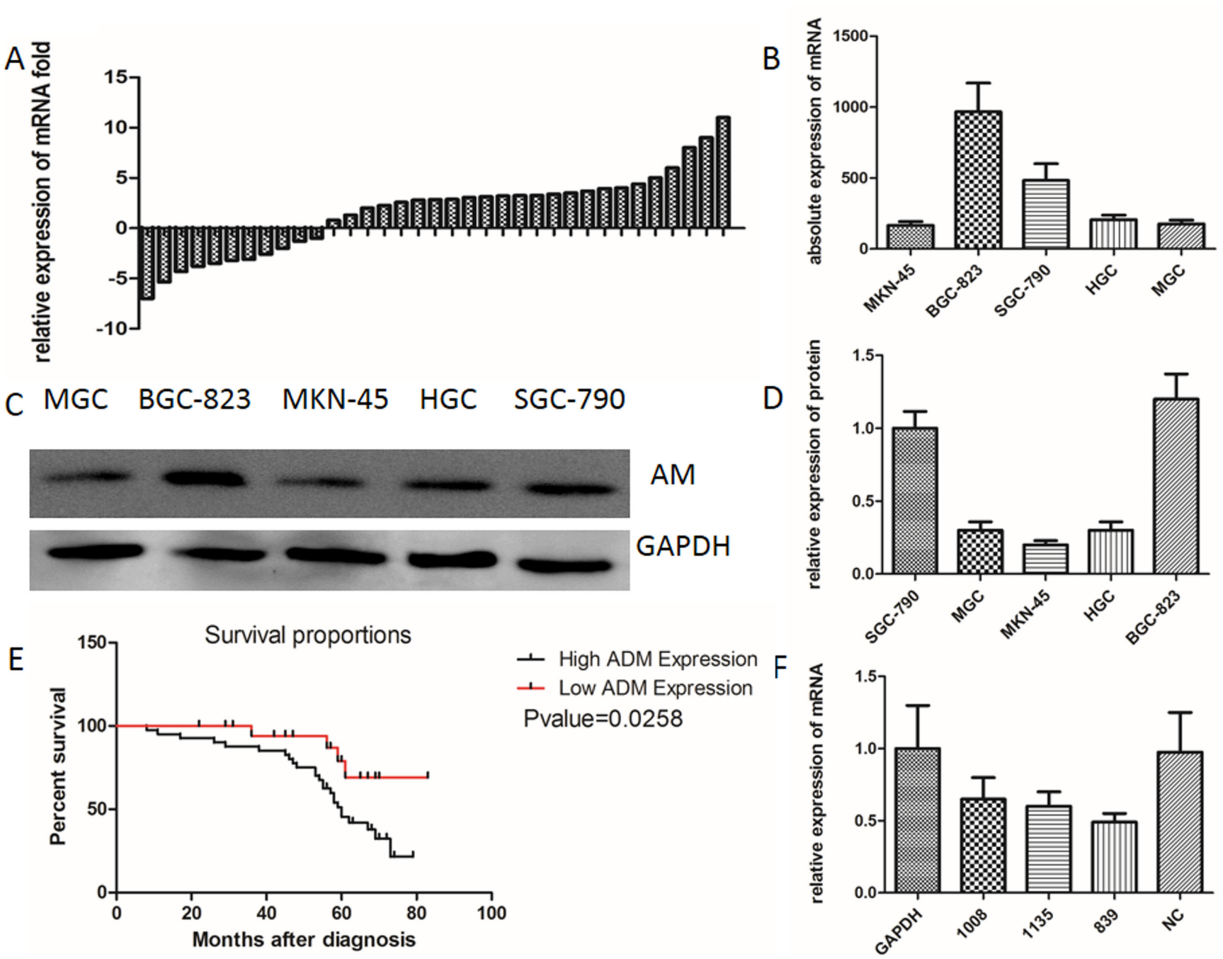
**(A)** qRT-PCR results showing the AM mRNA levels in 35 gastric cancer specimens. **(B)** Relative AM mRNA levels in the gastric cancer cell lines examined. **(E)** Survival analysis of the 60 clinical patients (P-value=0.0258). **(C)** Western blot showing the expression level of AM in the gastric cancer cell lines BGC-823, MKN-45, SGC-790, HGC, and MGC. **(D)** Gray-scale scanning histogram of the **(C)**. **(F)** qRT-PCR AM mRNA levels in transfected BGC-823 cells.

Our results indicate, that in 65% of the gastric cancer samples examined, AM protein levels were elevated as compared to the adjacent, non-tumor gastric tissues (Figure 1A). Furthermore, the levels of the AM receptors RAMP1, RAMP2, RAMP3, and CRLR were also elevated as compared to their adjacent non-tumor gastric tissues by 60%, 61%, 58%, and 62%, respectively (Figure 1B-1E). The AM mRNA expression levels were analyzed in 35 gastric carcinoma specimens (Figure 2A), and of these 35 specimens, 24 exhibited higher expression levels, representing 68% of the total specimens examined. A comparative survival analysis of the 60 patients is shown in Figure 2E. Our results show that the expression levels of AM, CRLR, RAMP1, RAMP2, and RAMP3 are higher in gastric cancer tissues than in the adjacent non-tumor gastric tissues, and the patients with the lower expression levels of AM lived longer (p=0.0258). Table 1 illustrates the relationship between AM expression and the clinicopathological factors of the 60 patients. Compared to the gastric cancer samples with low expression (0% to 25%), those with high expression (75% to 100%) were found to have larger tumor size (P=0.008), higher tumor grade (P=0.012), and higher TNM stage (P=0.011).

**Table 1:** Clinicopathological features and the expression of AM in gastric cancer patients

Parameters	Group	AM				P value
		0%-25%	26%-74%	75%-100%	Total	
Age (years)	≤ 60	12	9	25	46	0.731
	>60	3	2	9	14	
Gender	Male	10	8	28	46	0.495
	Female	2	2	10	14	
Tumor size	T1-T2	5	5	6	16	0.008
	T3-T4	2	12	30	44	
Tumor grade	G1	12	5	6	23	0.012
	G2/G3	7	12	18	37	
Distant metastasis	Yes	5	6	10	21	0.716
	No	9	7	23	39	
TNM stage	I-II	5	7	7	19	0.011
	III-IV	3	8	30	41	

The high expression samples (75%-100%) and low expression samples (0%-25%) were evaluated using chi-square test.

### Gene silencing in BGC-823 cells using AM-siRNA

**Figure 3:**
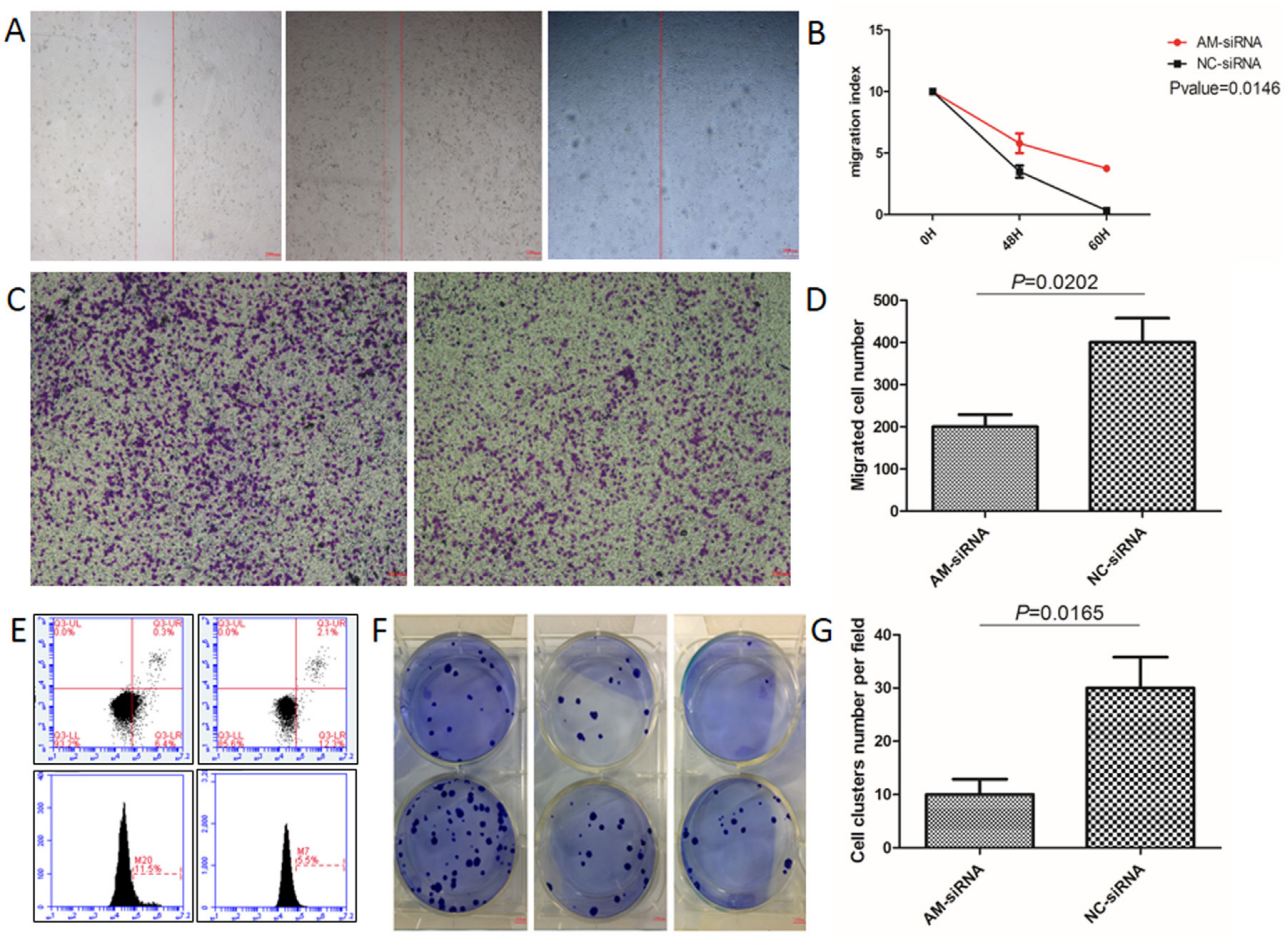
BGC-823 cells transfected with AM siRNA-839 **(A)** Wound healing assay (left, 0 h control group; middle, siRNA-839 60 h group; right, siRNA-GAPDH 60 h). **(B)** Quantitative analysis of **(A)** (P-value=0.0146). **(C)** Trans-well migration assay (left, siRNA-GAPDH experimental group; right, siRNA-839 control group). **(D)** Quantitative analysis of **(C)**. The data represent the mean ± SEM (P-value=0.0202). **(E)** Annexin V/PI staining (left, basic level of cell apoptosis; right, siRNA-839 cell apoptosis rate. **(F)** Colony forming assay (top, siRNA-839 experimental group; bottom, siRNA-GAPDH control group). **(G)** Quantitative analysis of **(F)**. The data represent the mean ± SEM (P-value=0.0165).

We selected the highest AM expressing specimens from the four different gastric cancer cell lines examined. Gastric cancer cell proteins were extracted from each of the cell lines, and western blot analyses were used to determine AM expression in the BGC-823 cell line (Figure 2C, 2D). qRT-PCR was used to determine the highest expression levels of mRNA in the BGC-823 specimens (Figure 2B), and the effects of the knockdown of AM via siRNA were identified (Figure 2F). The negative control, FAM, was used to monitor and optimize transfection efficiency. Annexin V/PI staining demonstrated that AM knockdown promotes apoptosis in BGC-823 cells (Figure 3E). A colony formation assay demonstrated that gastric cancer tumor growth was significantly decreased after the BGC-823 cells were transfected with AM siRNA (Figure 3F, 3G). The MTT cell proliferation assay showed no statistical significance. The wound healing assay and trans-well migration assay indicated that cellular migration activity decreased in response to the AM knockdown (Figure 3A-3D).

### AM regulates the AKT signaling pathway

**Figure 4:**
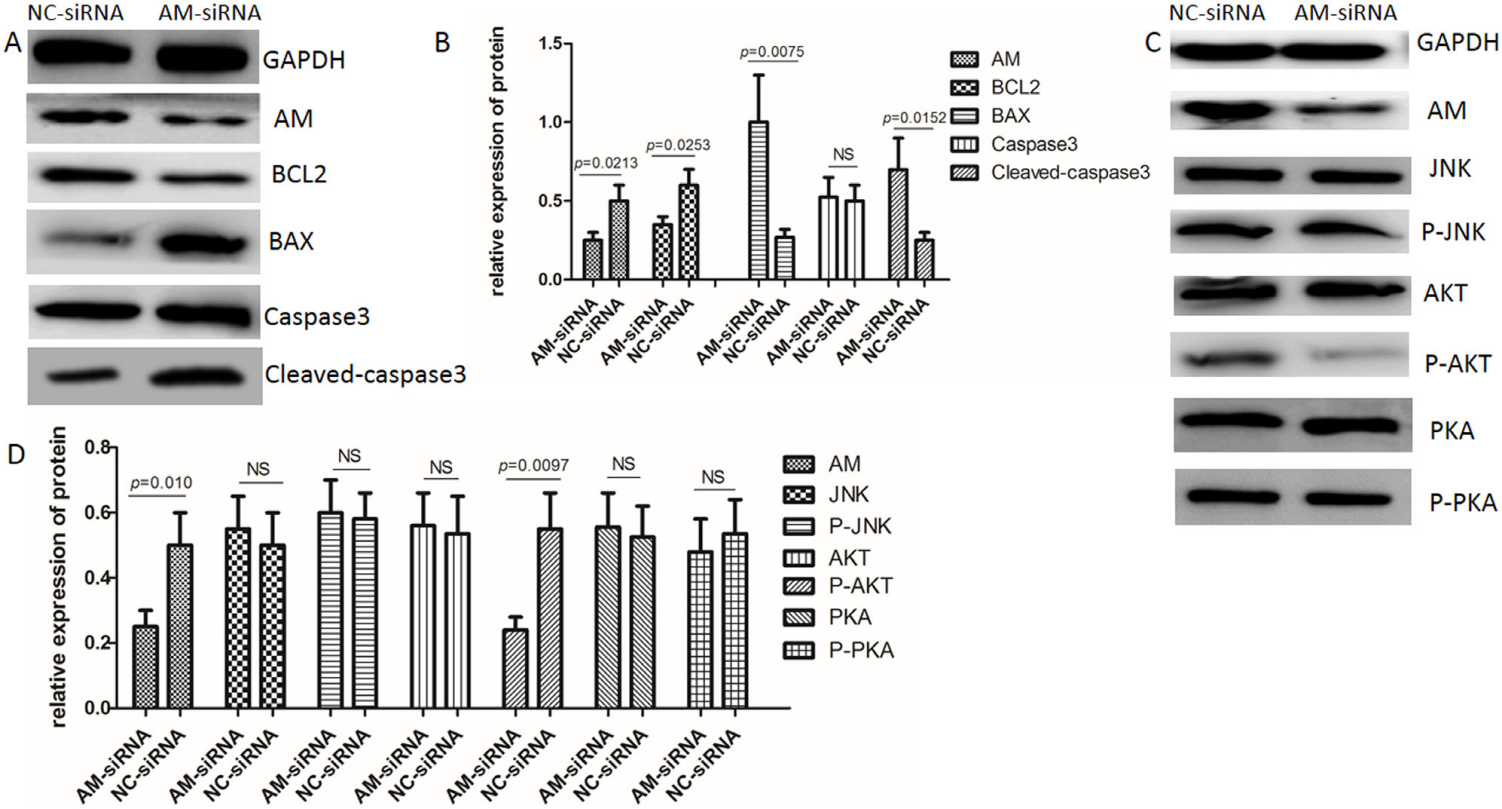
The AKT signaling pathway is down-regulated resulting in cell apoptosis **(A)** Western blot showing that Bcl2 levels are reduced while Bax and cleaved-caspase3 are elevated in response to AM knockdown. **(B)** Gray-scale scanning histogram of **(A)**. The data represent the mean ± SEM (P-value=0.0213; P-value=0.0253; P-value=0.0075; P-value=0.0152). **(C)** Western blot showing P-AKT levels are reduced in response to AM knockdown. **(D)** Gray-scale scanning histogram of **(C)**. The data represent the mean ± SEM (P-value=0.010; P-value=0.0097).

Our western blot analysis results showed P-AKT expression is reduced in response to reduced levels of AM in our knockdown assay, however, pronounced changes in P-PKA and P-JNK levels were not observed (Figure 4C, 4D). Furthermore, our results showed that AM-siRNA treatment might inhibit the Proteinkinase B (PKB, also known as Akt) signaling pathway and slightly suppress PKA activation. AM-siRNA activated the apoptosis mechanism. Our western blot results showed that Bcl2 expression was reduced, while cleaved-caspase3 and Bax expression was up-regulated (Figure 4A, 4B).

### HIF-1α regulates the expression of AM and VEGFA in the early stages of hypoxia, whereas NDRG3 regulates the late stages

**Figure 5:**
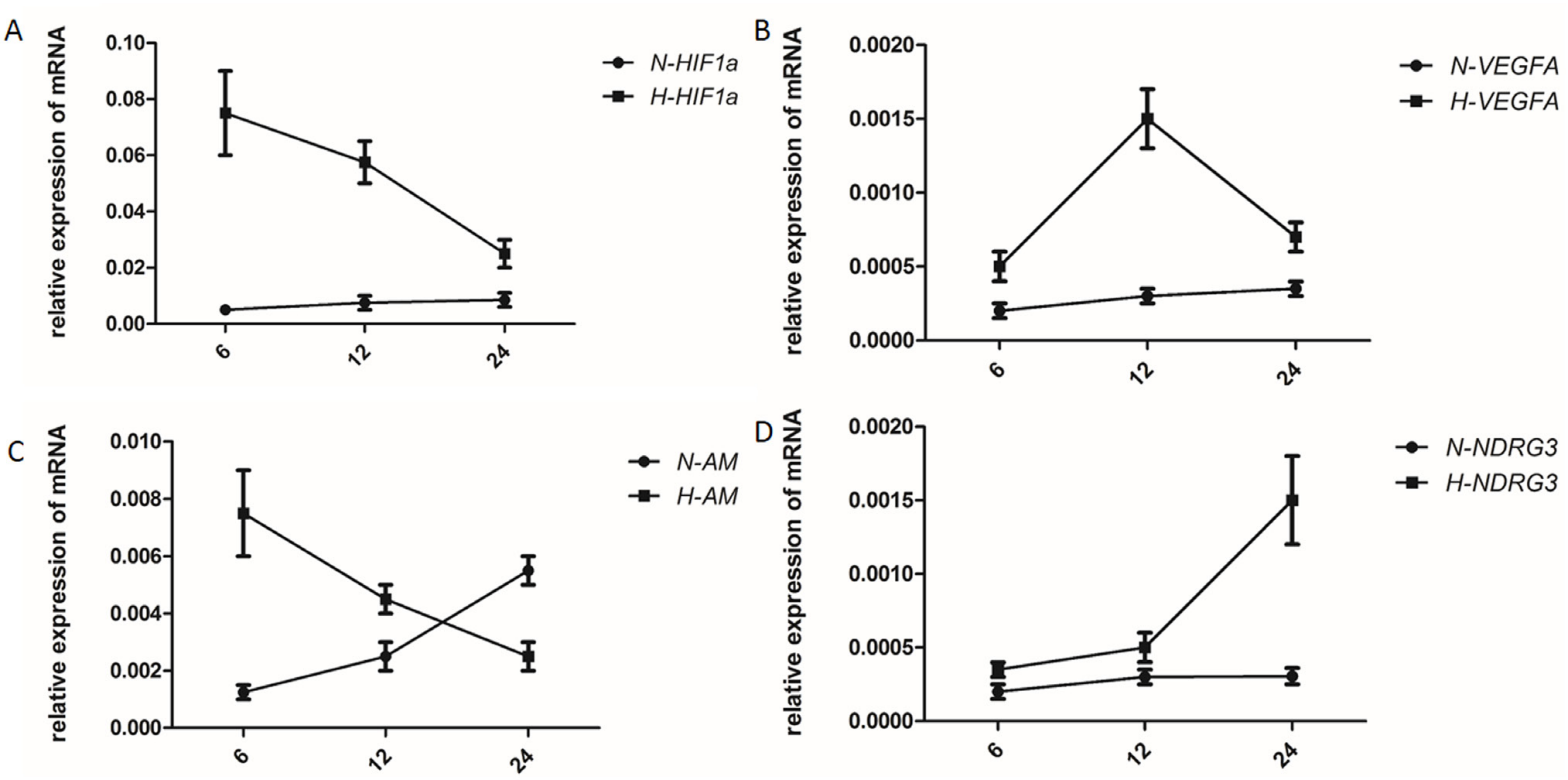
Induction of hypoxia in gastric cancer BGC-823 cells **(A)** Relative mRNA levels of AM, VEGFA, NDRG3, and HIF-1α following hypoxia treatment. The control group was treated using normal culture conditions in parallel. Charts **(A, B, C,** and **D)** show HIF1a, VEGFA, AM, and NDRG3, respectively. Expression levels of each target mRNA are expressed relative to GAPDH levels.

AM, VEGFA, NDRG3, and HIF-1αmRNA expression levels were measured under hypoxic conditions. Our qRT-PCR results showed the expression of AM, VEGFA, NDRG3, and HIF-1α allincreased, though to different degrees, and at different times, in the gastric cancer cell lines. After 6 h of culture, the expression of AM peaked (Figure 5C), whereas the expression of NDRG3 did not rise significantly until 24 h after exposure to hypoxic conditions (Figure 5D). Our results showed that HIF-1α regulates the expression of the AM and VEGFA genes in the early stages of hypoxia, whereas NDRG3 regulates the later stages.

### AM does not have a role in the regulation of autophagy in BGC-823 cells

**Figure 6:**
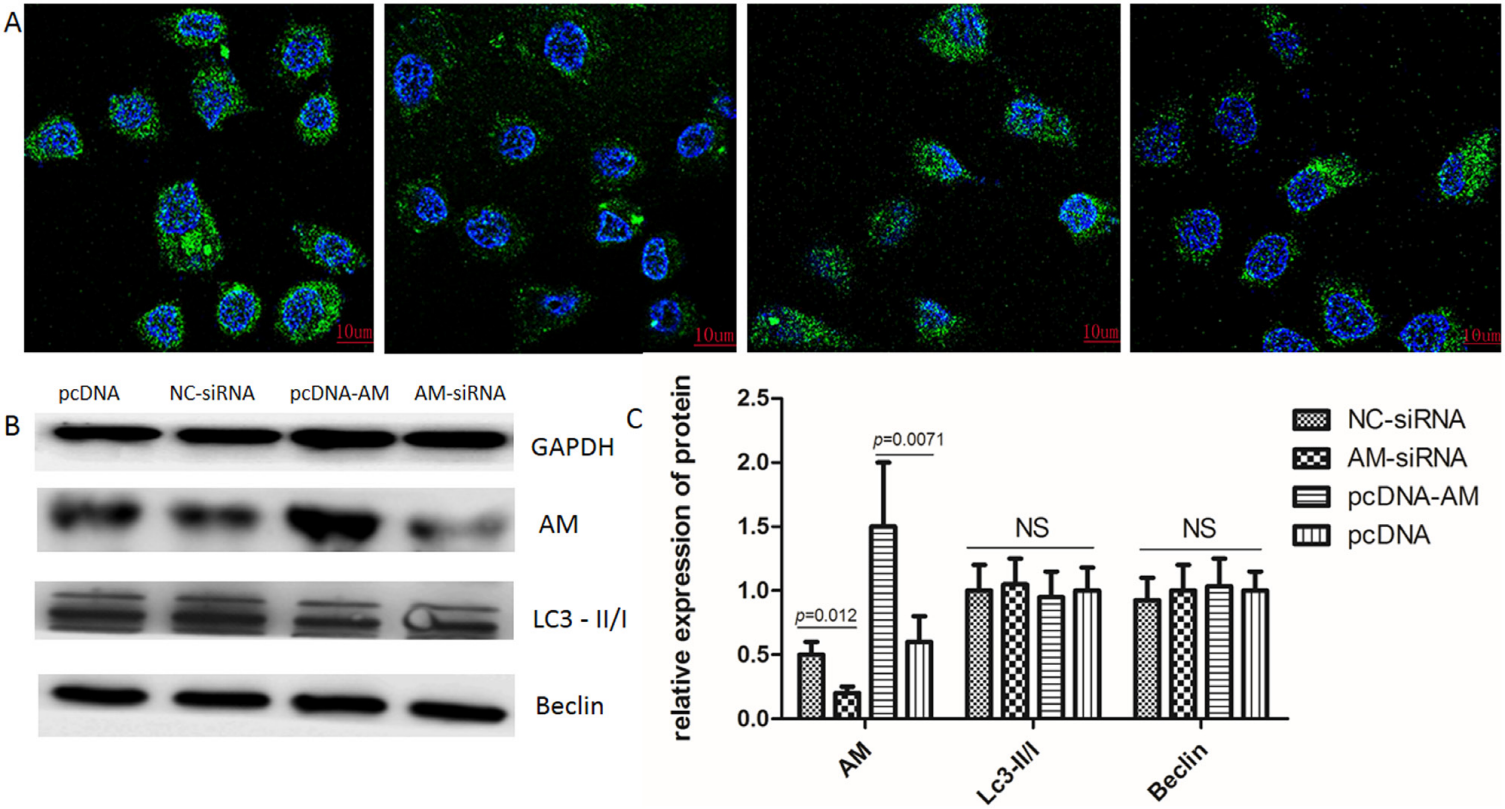
GFP-LC3 single fluorescent indicator autophagy **(A)** Tracer auto-phagosome formation. The images show the results of transfection with pcDNA and pcDNA-GFP–LC3, NC-siRNA and pcDNA-GFP–LC3, pcDNA-AM and pcDNA-GFP–LC3, and siRNA-839 and pcDNA-GFP-LC3 vectors, respectively. **(B)** Western blots showing the LC3-II/I ratio and Beclin levels were not changed. **(C)** Gray-scale scanning histogram of **(B)**. The data represent the mean ± SEM (P-value=0.0120; P-value=0.0071).

Western blots were used to determine the changes in the LC3-II/I ratio and Beclin protein levels to evaluate autophagy. No significant changes in theLC3-II/I ratio or Beclin protein levels were observed (Figure 6B, 6C). Our confocal microscope tracer autophagosome formation results showed that the BGC-823 cells, transfected with AM-siRNA and pcDNA-AM, had no effect on the rate of autophagy.

## DISCUSSION

The expression of AM and AM receptors has been shown to be elevated in human lung, colon, ovary, breast, bone marrow, prostate, and cartilage cancer cell lines [26]. Furthermore, in melanomas, the expression levels of AM, CLR, RAMP2, and RAMP3 have been shown to be higher than those in the control tissues [27]. In this study, we found that the levels of the AM protein and its receptors CRLR, RAMP1, RAMP2, and RAMP3 were also significantly increased, as compared to the adjacent healthy gastric tissues. In colorectal carcinomas, a correlation between high AM mRNA expression and protein levels has also been demonstrated [28–30]. Furthermore, AM and RAMP mRNA levels have been shown to be higher in pancreatic adenocarcinoma tissues, skin carcinomas, and pheochromocytomas, as compared to normal pancreatic tissue [27, 31, 32]. However, our results are not consistent with those of the above-mentioned previous studies; therefore, further investigation is necessary.

Malignant cancer cells have been shown to have elevated growth signal levels, angiogenesis and metastasis, and to inhibit apoptosis [33, 34]. Our results show that BGC-823 cells transfected with AM siRNA have significantly decreased gastric cancer growth and cellular migration activity. Our flow cytometry results indicate that AM knockdown promotes apoptosis in gastric cells. Previous studies have reported similar results, showing RNA interference targeting of AM induces apoptosis and reduces growth in human bladder urothelial cell carcinomas [22], and that AM is a potential therapeutic target in the treatment of colorectal cancer [28]. Furthermore, several studies have shown that AM receptors are effective targets in the treatment of pancreatic cancer [35]. Our results suggest that RNA interference-mediated silencing of AM suppresses gastric tumor growth, and may represent a novel strategy for the treatment of gastric cancer.

When tumor cells are exposed to hypoxic conditions, HIF-1α up-regulates several genes promoting the growth and survival of tumor cells [36, 37]. This response to hypoxia results in the accumulation of lactate [38], which enables the formation of the tumor microenvironment. Under hypoxic conditions, the expression of AM, HIF-1α, NDRG3, and VEGFA are significantly elevated in the human gastric cancer cell line BGC-823. Under similar conditions, human colorectal carcinoma cells have also been shown to display a time-dependent increase in AM mRNA and peptide expression [28]. Alternatively, the expression of AM and NDRG3 has been shown to not be time independent. AM is known to be involved in the early stage of the cellular mechanism of oxygen sensing, whereas NDRG3 is involved in the late phase [38]; moreover, AM is involved in the regulation of the tumor microenvironment. Therefore, AM may be involved in microvessel proliferation and partially in the release of hypoxia in solid tumors.

Autophagy is an evolutionary conserved catabolic process by which cells are degraded and recycled to maintain cellular homeostasis [23]. Several reports have found a connection between autophagy and apoptosis, and the regulation of starvation-induced autophagy by the phosphorylation of Bcl-2 [39, 40]. Furthermore, some studies have shown the regulation of autophagy is altered by the calcium signalosome in human diseases [23]. Other studies have demonstrated that AM can induce the mobilization of Ca^2+^ independent of cAMP levels [25]. In the present study, we used western blot analysis to detect changes in the LC3-II/I ratio, alongside the Beclin protein, to evaluate autophagy. Tracer autophagy bodies were examined, using a confocal laser scanning microscope; however, no significant changes were found. Therefore, further research examining the relationship between AM and autophagy is required.

In conclusion, our results indicate that AM plays a significant role in the proliferation, migration, and angiogenesis of gastric cancer, and RNA interference-mediated knockdown of AM and AM receptors may provide a novel treatment strategy for gastric cancer and/or solid tumor.

## MATERIALS AND METHODS

### Tissue specimens and patient clinical information

In total, 120 stomach tissue samples were collected and embedded in paraffin (FFPE) in accordance with the Ethics Committee of the School of Medicine (Jiangsu University) guidelines. Two samples were obtained from each of 60 patients diagnosed with adenocarcinoma: one cancerous tissue sample and one adjacent non-cancerous tissue sample. All tissue blocks were obtained from the Department of Pathology at the Affiliated Hospital of Jiangsu University between 2010 and 2015. None of the patients included in this study had received treatment (radiation therapy, chemotherapy, or immunotherapy) before surgical resection. The clinicopathological characteristics of the patients are presented in Table 1.

### Cell culture and reagents

Human gastric cancer cell lines, including BGC-823, MGC, SGC-790, HGC, and MKN-45 were purchased from the American Type Culture Collection in 2015. All cells were cultured in DMEM, supplemented with 10% fetal bovine serum, and were grown in a humidified atmosphere containing a 95% air, 5% CO2 mixture at 37°C. Anti-AM, anti-P-JNK, anti-JNK, anti-P-PKA, anti-PKA, and anti-LC3-II/I antibodies were obtained from Abcam Technology (Cambridge, MA). Anti-cleaved-caspase3, anti-caspase3, anti-P-AKT, anti-AKT, anti-Beclin, and anti-CRLR antibodies were obtained from Santa Cruz Biotechnology (Santa Cruz, CA). Anti-GAPDH, anti-Bax, anti-Bcl2, anti-RAMP1, anti-RAMP2, and anti-RAMP3 antibodies were obtained from Cell Signaling Technology (Beverly, MA).

### Immunohistochemical staining

The cell lines were treated with primary antibodies against AM (1:200), CRLR (1:100), RAMP1 (1:100), RAMP2 (1:100), and RAMP3 (1:100) at 4°C overnight. A 1:500 dilution of the corresponding peroxidase-conjugated IgG antibody was incubated with a streptavidin-peroxidase complex, using the Vectastain Elite ABC Universal kit (Vector Laboratories, Burlingame, CA) for 30 min at room temperature, and developed using a 3,3′-diaminobenzidine substrate. The specimens were counterstained with Gill’s hematoxylin, and dehydrated using ethanol before being fixed with xylene and subsequently mounted. The AM, CRLR, RAMP1, RAMP2, and RAMP3 stained cells were counted, in 5 randomly selected fields, on each slide at 400× magnification. The intensity of AM and AM receptor staining in the gastric cancer tissue samples was scored as 0% (negative), 25% (weak), 50% (moderate), 75% (strong), or 100% (strongest), as determined by a pathologist from ADICON CLINICAL LABORATORIES.

### Gene silencing in the BGC-823 cell line using AM-siRNA

BGC-823 cells were seeded to 6-well plates (2.5×10^5^ cells/well) and allowed to adhere for 24 h before transfection. Using Lipofectamine 2000 (Invitrogen, Groningen, Netherlands), the cells were transfected with siRNAs directed against human AM (AM-siRNA; GenePharma, Shanghai, China) or non-targeted control siRNAs (NC-siRNA; GenePharma). FAM (GenePharma) was used as a negative control to monitor and optimize transfection efficiency.

### Induction of hypoxia in gastric cancer cells

BGC-823 cells were seeded to 6-well plates, at a density of 3×10^5^ cells/well, to facilitate an exponential growth rate. The assay was then divided into six treatments: 6 h hypoxia, 6 h non-hypoxia, 12 h hypoxia, 12 h non-hypoxia, 24 h hypoxia, and 24 h non-hypoxia. Each treatment was replicated three separate wells. Each hypoxia treatment was exposed to 1% O_2_, 5% CO_2_, and 94% N_2_, at 37°C, for the designated period, and the non-hypoxia treatments were placed in a humidified atmosphere with a 95% air, 5% CO_2_ mixture at 37°C. Twenty-four hours after treatment, the cells were digested with trypsin, and total RNA and protein were extracted for analysis.

### Immunoblot analysis

Cell samples were washed in cold PBS, lysed for 20 min on ice with lysis buffer, and centrifuged at 14,000 *g* for 10 min at 4°C. The supernatant was collected and the protein concentration was determined using the Bradford assay (Bio-Rad Laboratories, Hercules, CA, USA). Total protein was separated on a 10% SDS–polyacrylamide gel and transferred to a PVDF membrane (Bio-Rad). The membranes were blocked with 5% skimmed milk for 1 h and incubated overnight at 4°C with the following primary antibodies: anti-AM, anti-P-JNK, anti-JNK, anti-P-PKA, anti-PKA, anti-Bax, anti-Bcl2 anti-caspase3, anti-Beclin, anti-cleaved-caspase3, and anti-LC3-II/I diluted 1:1,000, and anti-P-AKT, anti-AKT, and anti-GAPDH diluted 1:2,000. The membranes were then incubated with a secondary antibody for 1.5 h at room temperature. Bound antibodies were visualized using a chemiluminescent substrate (ECL, Amersham, Arlington Heights, IL)

### RNA extraction and real-time quantitative reverse transcription-PCR (qRT-PCR)

Total RNA was extracted from BGC-823 cells using the RNA iso Plus Kit (Takara, Dalian, China). Between 10 pg and 1 μg total RNA was used for first-strand DNA synthesis using the PrimeScript RT Reagent Kit (Takara), and qRT-PCR was performed using the Top Green qPCR SuperMix and Passive Reference Dye (Takara). The following primers were used for amplification: AM, forward primer (5′-CACTTCGGGCTTCTCACTGC-3′) and reverse primer (5′-ACATCAGGGCGACGGAAAC-3′) TM value: 60°C; VEGFA, forward primer (5’-AGGCCAGCACATAGGAGAGA-3′) and reverse primer (5′-TTTCTTGCGCTTTCGTTTTT-3′) TM value: 60°C; HIF-1α, forward primer (5′-GAAAGCGCAAGTCCTCAAAG-3′) and reverse primer (5′-TGGGTAGGAGATGGAGATGC-3′) TM value: 60°C; NDRG3, forward primer (5′-GACAAGCGCGCAGTCTCAAG-3′) and reverse primer (5′-TGAGTAGGAGTGCGAATCCGC-3′) TM value: 58°C; and GAPDH, forward primer (5′-TTGGTATCGTGGAAGGACTCA-3′) and reverse primer (5′-CAGTAGAGGCAGGGATGATGT-3′) TM value: 60°C. The annealing temperature was optimized by gradient PCR for each primer pair. The relative expression of each target mRNA relative to GAPDH mRNA was calculated using the ΔΔCt method.

### Flow cytometry

BGC-823 cells were transfected with AM siRNA after 48 h of cell growth and flow cytometry was used to detect apoptosis. The cells were stained with Annexin V-FITC and PI using an Apoptosis Detection Kit (Sigma-Aldrich, St. Louis, MO) according to the manufacturer’s instructions. BGC-823 cells transfected with FAM siRNA were used as the negative control group. The cells were counted with a FACS Calibur flow cytometer.

### GFP-LC3 single fluorescent autophagy indicator system

The pcDNA-GFP-LC3 single fluorescent autophagy indicator system was constructed as follows. AM-siRNA and pcDNA-GFP-LC3 vectors were used to transfect BGC-823 cells and transfected pcDNA-AM and pcDNA-GFP-LC3 vectors were used as controls. After 48 h of culture, western blots were used to detect LC3-II/I ratio changes in the Beclin protein to evaluate autophagy. The cells were counter-stained with DAPI 40727ES10 (Beyotime Institute of Biotechnology) for 20 min. Stained specimens were imaged using a confocal laser scanning microscope (Olympus).

### Statistical analysis

The *in vitro* experimental data depicted represent at least three independent experiments. Data are expressed as the means of the three or more independent experiments’ 6 standard error of the mean (SEM). The high expressing samples (AM: 75% to 100%) and low expressing samples (AM: 0% to 25%) were evaluated using the chi-square test. Significant differences between controls and treated samples were evaluated using one-way analysis of variance (ANOVA) with SPSS software. The results were considered to be statistically significant if the p value was less than 0.05. Data were compared using GraphPad Prism 5 software.
